# What is a unit of nature? Measurement challenges in the emerging biodiversity credit market

**DOI:** 10.1098/rspb.2024.2353

**Published:** 2024-12-11

**Authors:** Hannah S. Wauchope, Sophus O. S. E. zu Ermgassen, Julia P. G. Jones, Harrison Carter, Henrike Schulte to Bühne, E. J. Milner-Gulland

**Affiliations:** ^1^ School of GeoSciences, University of Edinburgh, Edinburgh, UK; ^2^ Department of Biology, University of Oxford, Oxford, UK; ^3^ School of Environment and Natural Sciences, Bangor University, Bangor, UK; ^4^ Department of Biology, Utrecht University, Utrecht, Netherlands; ^5^ Institute of Zoology, Zoological Society of London, London, UK

**Keywords:** credits, fungible, additionality, leakage, quantify, contribution

## Abstract

Bending the curve of biodiversity loss requires the business and financial sectors to disclose and reduce their biodiversity impacts and help fund nature recovery. This has sparked interest in developing generalizable, standardized measurements of biodiversity—essentially a ‘unit of nature’. We examine how such units are defined in the rapidly growing voluntary biodiversity credits market and present a framework exploring how biodiversity is quantified, how delivery of positive outcomes is detected and attributed to the investment and how the number of credits issued is adjusted to account for uncertainties. We demonstrate that there are deep uncertainties throughout the process and question if the benefits of biodiversity credits, and other efforts to abstract nature to a single unit, outweigh the harms. Credits can only be positive for biodiversity if they are used with unprecedentedly strict regulation that ensures businesses mostly avoid negative impacts and if they are purchased to quantify positive contributions rather than as direct offsets. While there may be a role for markets in attracting conservation funding, they will only ever be part of the solution, especially for the many aspects of nature that cannot be reduced to a unit.

## A perspective on the inaugural Georgina Mace Review

It was a great honour to be asked to write the inaugural Georgina Mace Review for *Proceedings B*, but also quite daunting. What topic could do justice to her incisive contributions that cut through muddled thinking, and laid the scientific foundations for international conservation policy and practice? It should be something that builds on her body of research within biodiversity science. It should also engage with some of the things that her work epitomised: clarity of thought and language, practical useability of the approaches suggested, and robust underlying science. It rapidly became clear that there was one topic that fitted this brief; how biodiversity gains and losses are being measured to inform international action towards nature recovery. This is highly salient to policy and practice right now, a field in which biodiversity science has useful contributions to make, and a topic that Georgina made foundational contributions to.

Redesigning the IUCN Red List of Threatened Species was Georgina’s first, and huge, contribution to international conservation policy and practice. She started by deploying her and her co-author Russ Lande's understanding of population genetics and ecological modelling, to set out a clear and simple framework for thinking about extinction risk [1]. This led on to a series of workshops, which I was lucky to be involved in as a very early career post-doc, thinking through how these scientific principles could translate into practical criteria for assigning species to categories of extinction risk. The challenge was devising criteria which were applicable to all eukaryotic organisms and were feasible to implement even when data on a species were minimal. This was a labour of love, over years, for a small group of people led by Georgina, which grew into an enormous red listing effort by the global network of thousands of scientists that is the IUCN Species Survival Commission. The Red List has become the standard for assessing the threat status of a species (see https://www.iucnredlist.org/). As Georgina and some of the colleagues who were most involved throughout the process reflected in a chronicle of the process [2], it also taught us a lot about how to develop, test and refine metrics and standards in a way that is inclusive, global and able to incorporate wide expertise. The lessons learnt in the development of the Red List were taken on board in two recent IUCN products: the Green List of Protected and Conserved Areas [3] and Green Status of Species [4].

Georgina was engaged with some of the most influential groups working to underpin international conservation policy with science, such as the Intergovernmental Panel on Biodiversity and Ecosystem Services [5]. Closer to home, the UK’s National Ecosystem Assessment was a pioneering stocktake which not only provided an overview of the state of biodiversity in the UK to support policy-making; it set biodiversity within a broader social-ecological systems framing, explained complex concepts in ways that a broad readership could understand, and provided insights on ways forward towards sustainable futures [6]. Georgina’s collaborations with economists in this initiative sparked new ways of thinking [7]. Her work often brought into focus topics where woolly thinking was hindering progress; for example she wrote the definitive, clarifying, explanation of how biodiversity should fit within the framing of ecosystem services [8].

International conservation policy largely revolves around the Convention on Biological Diversity, and Georgina has made a substantial contribution in this space. For example, she highlighted the need for better targets, better monitoring and better indicators (e.g. [9]). This directly influenced the Aichi targets, which came into force in 2010 and were an attempt to define quantitative aims for signatory governments: a milestone in enabling scientists to contribute approaches for tracking progress and to deploy these in holding governments accountable. As the Decade of Biodiversity within which the Aichi targets were operational (2010–2020) came to an end, Georgina and colleagues outlined what was needed to go beyond the limited ambition of the Aichi targets to slow the rate of biodiversity loss in the next iteration of biodiversity targets. She called for action to 'bend the curve' for biodiversity; reversing the trend of nature loss towards nature recovery [10]. She was part of a team that went on to demonstrate that this can be done, if we combine both traditional conservation (protected areas) with reform of systems of production and consumption [11].

The Kunming–Montreal Global Biodiversity Framework was agreed by more than 190 countries in December 2022. The 'bending the curve' ambition that Georgina championed made it into this Framework, in the 2030 mission to 'halt and reverse' the loss of biodiversity. Wider society, including business and government, are making efforts to fulfil this mission. Given that this requires 'net gain' in biodiversity—preservation of existing natural areas is not enough—impacts of human activity on nature must be compensated for if they can’t be avoided or reduced. This requires the calculus of biodiversity gain and loss which our inaugural Review interrogates within the specific domain of biodiversity credit markets. So, the Review builds directly on Georgina’s foundational research, and aims to provide useful, actionable, insights to support the many actors that are trying to implement this net gain calculus practically, in the real world, and in the presence of much uncertainty.

Georgina was a mentor to many; she actively sought out early career researchers to bring into the initiatives she led (as she did for me). She was also an advocate for interdisciplinary collaborations. So I hope she would have been particularly pleased to see that this inaugural Review is a collaboration of predominately early-career researchers, including people with backgrounds in ecology, economics and finance. This has been a very exciting paper to write, and I thank the Royal Society for the opportunity; I think and hope that it is a paper Georgina would have enjoyed being part of.

*Professor Dame E. J. Milner-Gulland*

## 1. Introduction

In 2018, Professor Dame Georgina Mace—the acclaimed scientist after whom this new review series is named—led a resonant call for international biodiversity policy: conservation should go beyond managing the decline of nature and instead ‘bend the curve of biodiversity loss’ [10]. This ambition was reflected in the Convention on Biological Diversity’s 2022 Kunming–Montreal Global Biodiversity Framework as a commitment to ‘halt and reverse biodiversity loss’ by 2030. Achieving this will require cooperation from the business, regulatory and financial sectors to both reduce impacts on biodiversity and increase spending to plug the considerable funding shortfall [12].

There is a consequent push towards developing standardized means of measuring biodiversity, whether to enable businesses to disclose their negative impacts and contributions towards positive impacts in consistent ways, to enable regulators to set outcome-based biodiversity targets, to commodify biodiversity for emerging financial mechanisms or to assess progress towards the Global Biodiversity Framework’s goals [13]. However, biodiversity is neither one entity nor valuable for one reason, making meaningful generalized measurements—that consistently reflect biodiversity’s status and value in different settings—a philosophical challenge [14,15]. There are also technical challenges associated with the capability of current technologies to measure biodiversity across scales, and in demonstrating the cause of any measured unit of biodiversity gain or loss.

Nonetheless, efforts continue apace to develop standardized metrics that measure loss or gain in units of nature (note that although the terms ‘biodiversity’ and ‘nature’ are often used interchangeably, ‘biodiversity’ refers specifically to the variability of living organisms while ‘nature’ is a broader concept encompassing non-living elements such as landscapes and watercourses). In particular, there has been an explosion of actors developing biodiversity credits, a new asset class designed to entice investment into conservation. The methodologies being developed by biodiversity credit organizations provide an opportunity to explore and expose how the challenges in defining units of nature are being addressed.

A biodiversity credit is a certificate that aims to represent a measured and evidence-based unit of positive biodiversity outcome that is additional to what would have otherwise occurred [16]. Credits may be acquired for a range of reasons such as demonstrating that investment in conservation has had an impact, addressing a firm’s exposure to nature-related risks or as evidence that the firm is contributing towards positive biodiversity outcomes. The hope is that a market selling biodiversity outcomes as discrete units will remove barriers for businesses wanting to invest in nature by reducing complexity and improving transparency in measuring outcomes [16,17]. Proponents argue that this has the potential to greatly increase finance for conservation.

It is worth clarifying the relationship between the terms ‘biodiversity credit’ and ‘biodiversity offset’. Biodiversity offsets, which have existed since the 1970s [18], aim to achieve a minimum ‘no net loss’ of biodiversity, by compensating losses from a development with gains elsewhere, after having implemented previous steps of the mitigation hierarchy (avoiding and minimizing impacts and restoring onsite [19]). Offsets may be produced either by protecting threatened biodiversity (‘avoided loss’ offsets) or restoring degraded biodiversity (‘restoration’ offsets) [20]. Biodiversity offsets often involve the trading of units of biodiversity in a market [21] and can be voluntary or mandated by the government. Biodiversity offset markets are a dominant driver of private investment in conservation, generating an estimated US$11.7 billion investment in 2023 [22]. Debates have existed for decades about the best ways to quantify biodiversity for offsetting schemes given that this choice hugely influences outcomes [18,23,24].

Some industry-focused reports have distinguished the emerging voluntary biodiversity credit market from biodiversity offsets, by saying that credits are intended to finance gains for biodiversity which are not linked to negative impacts [17]. However, this distinction is somewhat artificial for two reasons. Firstly, if credits are used to make claims of net positive impacts on nature, these cannot be made without explicit reference to how negative impacts have been compensated for [19]. Secondly, the International Advisory Panel on Biodiversity Credits has laid out a set of models for how biodiversity credits markets could operate, which includes offsetting as a use-case [25]. This means that, in practice, voluntary biodiversity credits are likely to be used to offset harms. Somewhat confusingly, units traded under existing regulatory biodiversity offset schemes (such as Biodiversity Net Gain in the UK) are also sometimes termed biodiversity credits.

The voluntary biodiversity credit market faces an additional measurement challenge beyond what has been encountered so far in biodiversity offsetting schemes. In biodiversity offsetting, there have been limits to fungibility (the extent to which units are interchangeable between sites), with different measurement approaches in different systems. However, emerging voluntary biodiversity credit operators are attempting to define a unit of nature that is generalizable across regions and ecosystems or even across the globe. Yet, even with lesser measurement challenges, biodiversity offsetting schemes have been plagued with issues in demonstrating additionality (that benefits are beyond what would have happened in the absence of intervention), a lack of leakage (that impacts are not simply displaced to elsewhere in the landscape), and that there is some degree of permanence (that benefits persist) [26–28]. For example, multiple reviews have found that while the evidence base is small, the majority of evaluated biodiversity offsets did not achieve no net loss [29,30].

Similar issues have been faced by another nature-based market: forest carbon credits. These face lesser measurement challenges than biodiversity credits, in that there is a universally accepted unit of one tonne of CO_2_ (or CO_2_ equivalent). Yet, carbon credits have been strongly criticized for not often representing truly additional gains [31], a recent high-profile study highlighting this [32] contributed to US$1 billion being wiped off the value of the voluntary carbon market [33].

With this background, it is important to investigate how biodiversity credit operators are defining a unit of nature, and whether they can overcome the difficulties encountered in biodiversity offsetting schemes and the forest carbon market. Here, we review a sample of emerging methodologies from organizations proposing to sell biodiversity credits on the international voluntary market, categorize the decisions they make in defining a unit of nature and explore key challenges. We aim to provide those involved in marketing, evaluating or buying biodiversity credits with a better understanding of what is being sold. More broadly, we hope to give readers an understanding of the promises and perils of attempting to abstract biodiversity to standardized, fungible metrics and what this means for efforts to bend the curve of biodiversity loss.

## 2. Approach

We used the database provided in [34] and additional grey literature searches to identify biodiversity credit operators with detailed methodologies available as of 31 July 2024 (see electronic supplementary material, table S1 for a list of considered and reviewed methodologies). We focused on the majority of credit operators that sell credits based on biodiversity outcomes and did not review those that sell credits based on management action.

We categorized the stages methodologies go through (whether explicitly or implicitly) to create a biodiversity credit. These are: (i) framing (what does one credit represent), (ii) quantifying (how is biodiversity measured at the project site at any one point in time), (iii) detecting (how is conservation or restoration detected through time, and attributed to the investment), and (iv) adjusting (how are the number of credits issued adjusted to account for factors outside project control).

**Figure 1.**
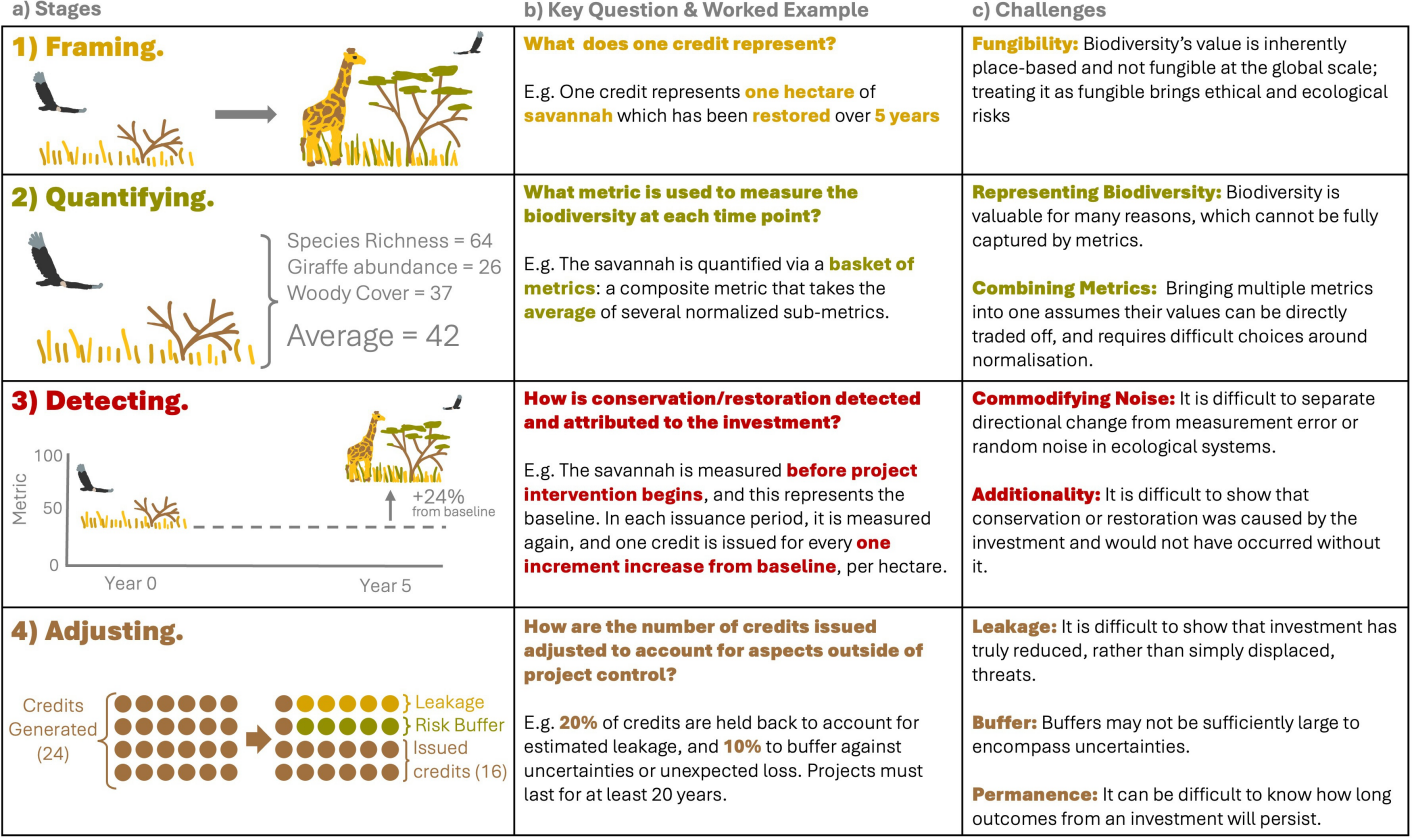
(*a*) The stages biodiversity credit methodologies go through to create a unit of nature that can be traded to generate revenue for conservation. Numbers in stage 2 ('quantifying’) are normalized measures of each metric. (*b*) The key question being answered at each stage, and a worked example for a hypothetical credit (note bold, coloured text could be replaced by another decision, see figures 2–5 and electronic supplementary material, tables S1–S5 for full details). (*c*) Key challenges associated with each stage.

In figure 1, we present an overview of these stages, walk the reader through a focal example, and highlight the key measurement challenges encountered at each stage. We now investigate each stage in turn. Given the rapid development of this field, it is likely that the details of methodologies will change, so we avoid naming specific ones in the text. However, we use superscript letters to direct the interested reader to electronic supplementary material, tables S1–S5, which give full details of the decision each methodology takes, as of August 2024.

**Figure 2.**
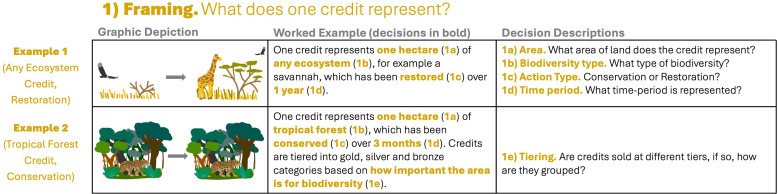
Decisions taken within the ‘framing’ stage (see figure 1). ‘Any ecosystem’ restoration credit (example 1) and a tropical forest conservation credit (example 2). Bold, coloured text in column 2 shows what decision has been taken by the example credit; this text could be replaced by other decisions depending on answers to the questions shown in column 3 (see electronic supplementary material, table S2 for more details).

**Figure 3.**
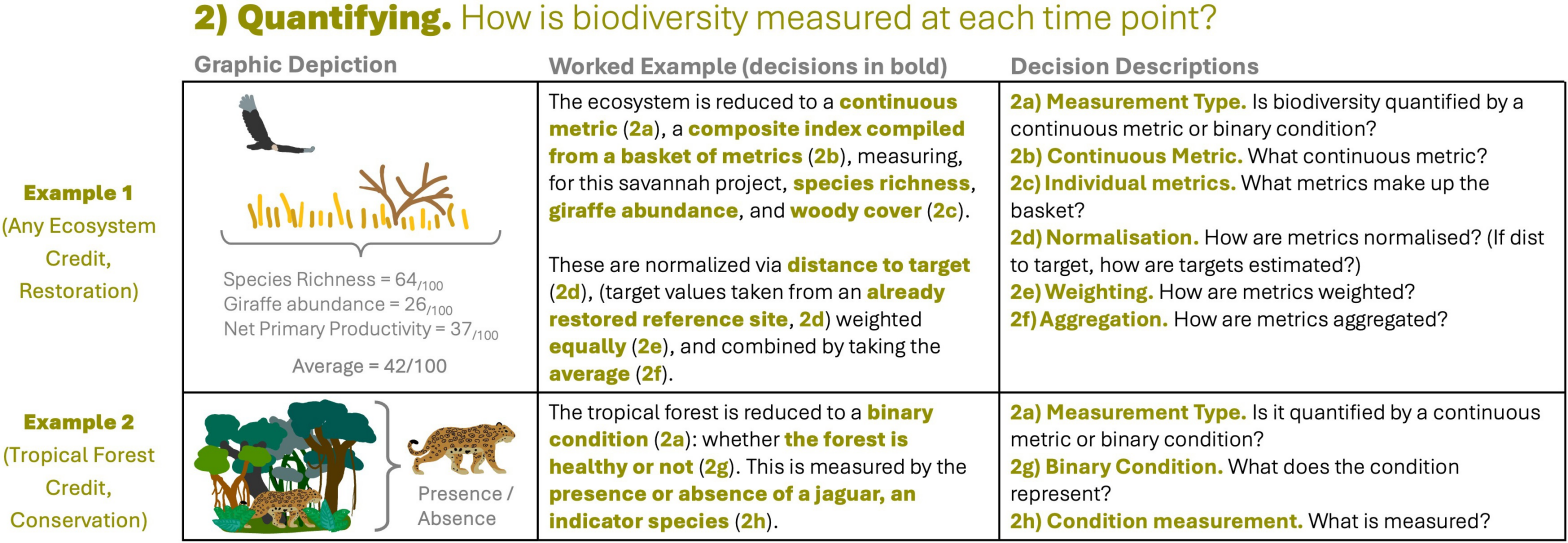
Decisions taken within the ‘quantifying’ stage (see figure 1). ‘Any ecosystem’ restoration credit quantified by a basket of metrics (example 1) and a tropical forest conservation credit quantified via a binary condition (example 2). Bold, coloured text in column 2 shows what decision has been taken by the example credit; this text could be replaced by other decisions depending on answers to the questions shown in column 3 (see electronic supplementary material, table S3 for more details).

**Figure 4.**
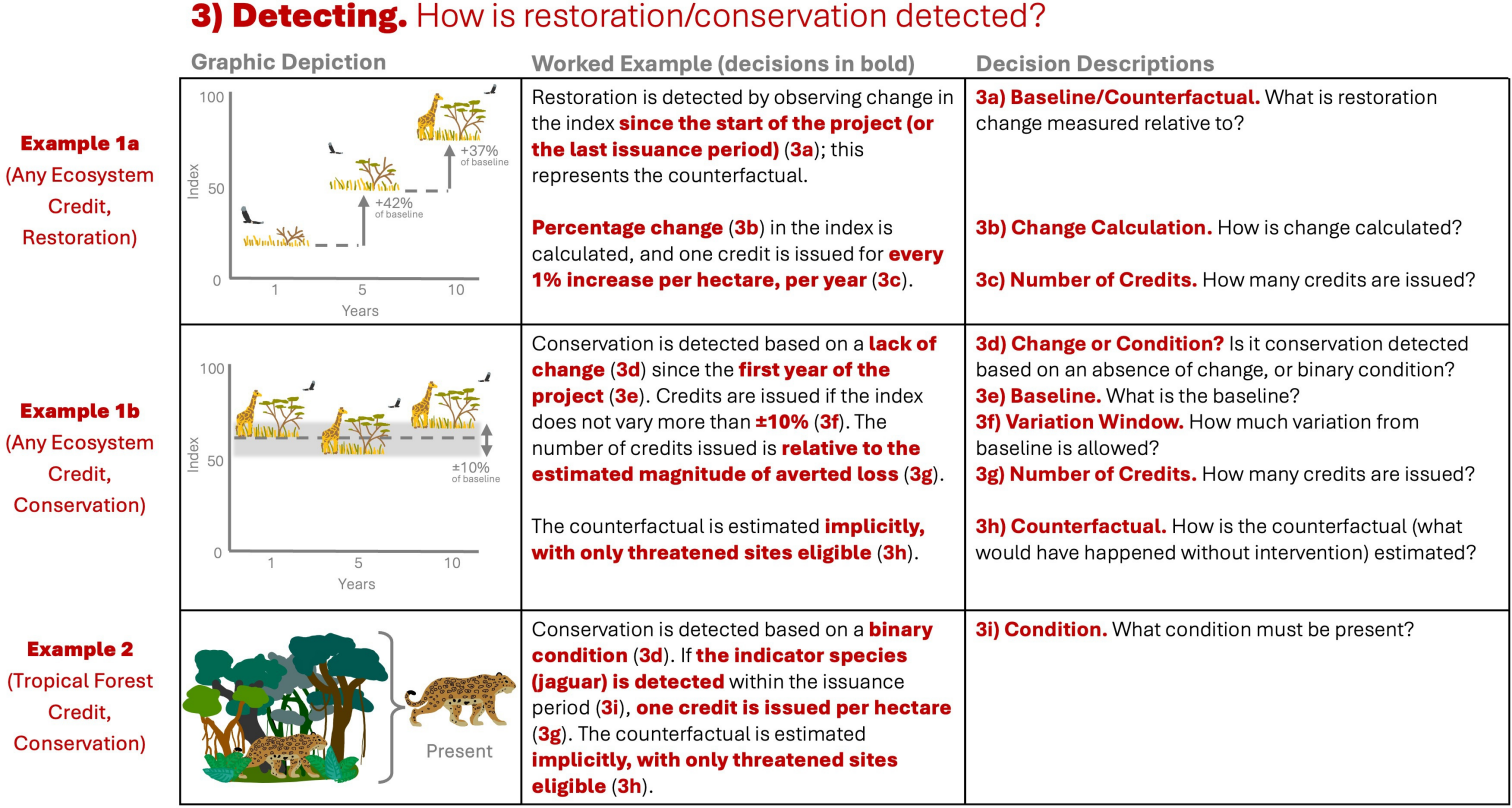
Decisions taken within the ‘detecting’ stage (see figure 1). ‘Any ecosystem’ credit, quantified continuously (example 1) and a tropical forest conservation credit quantified via a binary condition (example 2). Example 1 has been expanded into 1a (restoration) and 1b (conservation), to demonstrate how conservation and restoration are often detected when the ecosystem is quantified continuously. Bold, coloured text in column 2 shows what decision has been taken by the example credit; this text could be replaced by other decisions depending on answers to the questions shown in column 3 (see electronic supplementary material, table S4 for more details).

**Figure 5.**
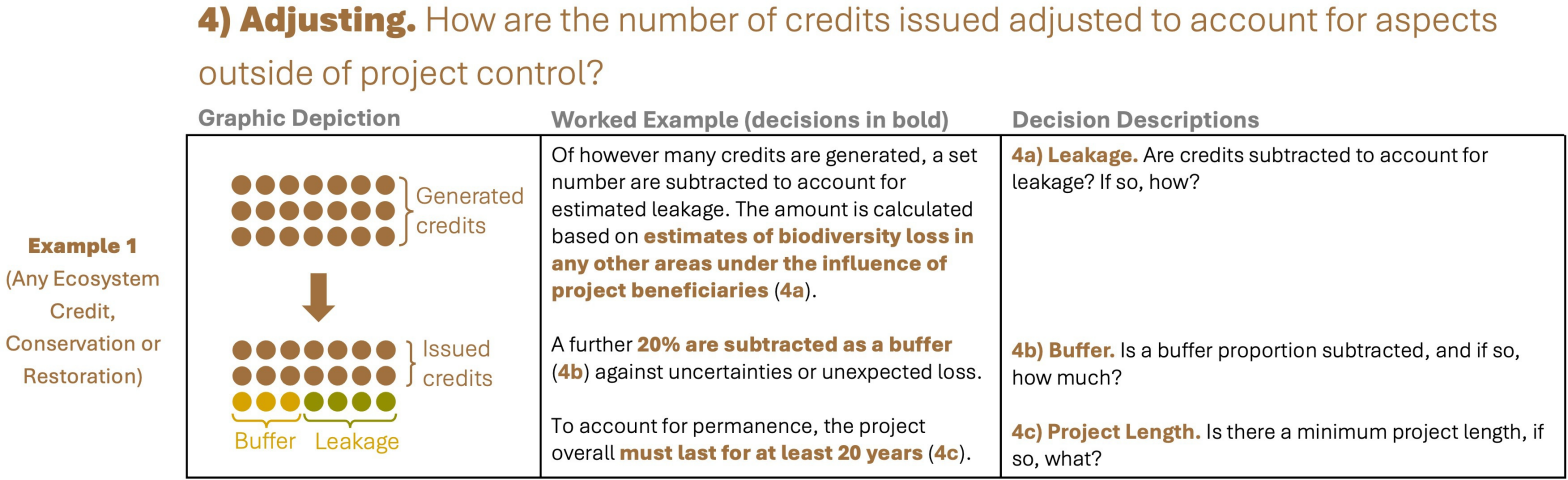
Decisions taken within the ‘adjusting’ stage (see figure 1). Showing just one example because adjusting decisions are agnostic to credit type. Bold, coloured text in column 2 shows what decision has been taken by the example credit, this text could be replaced by other decisions depending on answers to the questions shown in column 3 (see electronic supplementary material, table S5 for more details).

### (a) Stage 1: Framing

#### (i) Methodology decisions

‘Framing’ considers the decisions that define what kind of biodiversity a credit represents and over what time period. All methodologies we reviewed (except one^j^) sell credits by area; representing biodiversity in units of one hectare (figure 2; decision 1a). Methodologies specify what ecosystems they can be applied to, with many being designed to represent *any* ecosystem—terrestrial, marine or freshwater^c,e,f,g,h,i,k^. Others are more specific, to either terrestrial^a^ or marine^d^ biomes, to certain ecosystem types, for example, tropical forest^b,l^ or to certain characteristics of sites, for example, key biodiversity areas^f^ (figure 2; decision 1b).

A key distinction in credit types is whether they represent units of *conserved* (sometimes known as ‘avoided loss’ or ‘preservation’ credits) or *restored* (sometimes known as ‘uplifted’) biodiversity (figure 2; decision 1c). In most cases, the detection of the two is quite different (see §2c). Many methodologies give separate definitions for how to calculate each type^c,e,f,k^, though some are focused on just conservation^a,b,d,g,l^ or restoration^j^ or have the same method for both^h,i^.

Finally, methodologies define the time period that one credit represents (figure 2; decision 1d). For instance, one restoration credit might be issued for every 1% gain in biodiversity over a 5-year time period. Many credits represent 1 year^a,b,d,f^, although some represent time periods that are shorter, for instance, one month^g^, or longer, for instance, the total length of a project^i,j^.

In most cases, individual credits are designed to be sold at the same price, and if a project generates better outcomes—for instance, greater levels of restoration—more credits are issued. However, some methodologies also tier their credits, and sell some at premium prices based on other project inclusion criteria^c,f,g,h,l^ (figure 2; decision 1e). For instance, credits might be grouped into gold, silver and bronze tiers depending on whether projects occur in areas important for biodiversity.

#### (ii) Challenges

##### Fungibility

Fungibility—interchangeability of units—is key to commodifying biodiversity because, at least in theory, it allows efficient allocation of resources in a market [18,24]. However, biodiversity is multi-faceted and complex, meaning that fungibility is a social construct, not an ecological property. Many of the quantification approaches taken allow different metrics for different areas (see §2b), which goes some way to reflecting the fact that ecosystems can be important for different reasons. Yet, it still means that changes in measured quality in one ecosystem are rendered equal to changes in another. This ignores the place-based importance of biodiversity: the many ways in which different aspects make direct and indirect contributions to human well-being [8] depend on where the biodiversity is relative to people and how people relate to it [35]. Place is also important in terms of connectivity—an isolated patch of forest is not equivalent to one within a contiguous landscape [18]. To render any of these axes of value equivalent is to ‘omit, obscure or conceal’ them [36], and will mean resources directed to credits are unlikely to be allocated optimally for nature or people.

Despite this, most accept that rendering ecosystems fungible is necessary to some degree. Biodiversity offsetting markets generally establish trading rules that determine what types of nature are interchangeable, and across what distances. For example, in China, the national forest offset system allows any area of forest to be replaced by any other area, so long as it is also a forest [37]. In England’s Biodiversity Net Gain system, the trading rules allow a unit of habitat to be exchanged with one with the same or higher ‘distinctiveness category’, with a strong preference for ‘like for like’ trades as local as possible to the impact site [38]. These choices at least go some way to increasing the comparability of sites where biodiversity is gained or lost. In contrast, biodiversity credit methodologies designed to be applied to any ecosystem push fungibility to its extreme by attempting to render biodiversity gains equal across continents and biomes. Some operators tier credits to attempt to retain some of the masked value (decision 1e), however, this still means all credits within each tier are considered fungible.

### (b) Stage 2: Quantifying

#### (i) Methodology decisions

‘Quantifying’ considers the choice of metrics used to reduce the complexity of biodiversity at a site at any one time to a single value. There are two broad approaches taken to this (figure 3; decision 2a): those which assign a numeric value to the ecosystem(s) present at a site, where a higher number indicates higher biodiversity value, and those which classify the site according to a binary condition, reflecting some definition of healthy or not healthy. In a small number of cases, both approaches are used—a condition to decide if credits are issued at all and a numeric value used to assign worth if so.

Methodologies assigning numeric values almost always bring together multiple metrics into one value^a,c,d,e,f,h,i,k,l^. Some methodologies term this approach a ‘basket of metrics’^k^ (inspired by the Retail Price Index). In the academic literature, this approach is referred to as a composite indicator [39]—we continue to use ‘basket of metrics’ for consistency. The choice of metrics that go into the ‘basket’ varies greatly by methodology (figure 3; decision 2c), although most aim to capture a range of population, species and ecosystem-level metrics—for example, abundance of a target species, species richness and a measure of ecosystem structure (see example 1, figure 3).

Methodologies then face the difficulty all composite indicators face: how to bring together metrics measured on different scales into one value, which reflects the overall state of the system [40,41]. There are three steps to this: normalization—shifting metrics to the same numeric scale; weighting—assigning relative importance to each metric, and aggregation—bringing metrics together by, for example, taking the average.

Normalization is necessary, as otherwise, any metric with larger absolute values will have a disproportionate impact on the final composite indicator (figure 3; decision 2d). Many normalization procedures exist for scaling data that have already been collected [39], but these assume that the full range of possible values is represented in the data. In most cases, especially with restoration credits, this is not the case, as restoration should improve values beyond the initially measured range. For this reason, most methodologies normalize via a ‘distance to target’ approach in which metric values are scaled between 0 and 1, where 1 represents the ‘target’—an estimate of what the metric would be in a pristine ecosystem^c,d,e,h,k^. Most methodologies use linear scaling (e.g. if target species richness were 30 and the measured value was 15, the metric would be normalized to 0.5), though others allow for metric-specific scaling^c,e^. Of course, this approach means estimating the target value, i.e. what would the biodiversity metric be in a pristine or recovered ecosystem (figure 3; decision 2d). Most suggest using a range of techniques to estimate this, including reference to intact sites nearby, models, consultation with experts or the use of historical data.

The next step is weighting (figure 3; decision 2e)—how much should each metric contribute to the final composite indicator? Many methodologies do not use a system, thereby assuming equal weighting^a,f,h,i,k,l^, though some give certain metrics more importance^c,d,e^. Finally, methodologies decide how metrics will be aggregated (figure 3; decision 2f). Most use simple methods such as mean^h,c^, median^k^ or sum^a,d,f,i,l^.

In addition to, or instead of, the above decisions, some methodologies define a binary condition that marks the site as healthy or not^a,b,g,l^ (figure 3; decision 2g). Mostly, this condition is measured according to one metric^a,b,g^ (figure 3; decision 2h): for example, a site could be marked as healthy if deforestation has not occurred^b^, or if a sensitive indicator species is detected as present^a,g^.

#### (ii) Challenges

There are two key challenges within the ‘quantifying’ stage. Firstly, representing the value of biodiversity in a measurable way, and secondly, handling the inevitable trade-offs when multiple metrics are combined.

##### Representing biodiversity

When methodologies specify how to quantify biodiversity, they are implicitly stating what is valuable about it. Metrics necessarily focus on aspects of nature that *are* quantifiable, ignoring those that are either technically challenging or conceptually impossible to quantify, such as species interactions [42], the cultural value of nature, or its constitutive value as a part of the identities of Indigenous peoples and local communities [35,43].

Those methodologies that class sites into binary conditions define value in broad terms, for example, healthy/not healthy or intact/not intact. There are advantages in terms of simplicity and cost effectiveness, plus the benefit that it is relatively easy to understand what one credit represents [44]. However, they make the strong assumption that what is measured acts as a proxy for condition, which may not always be the case: biodiversity value is unlikely to be fully represented by one or a few umbrella species [45], and sites can be degraded in many ways short of outright structural habitat destruction [46]. Those methodologies that assign numeric values to the quality of a site are more explicit, and more complex, in defining what they find valuable about a site, but face further challenges in terms of how metrics are combined.

##### Combining metrics

Combining metrics presents two distinct challenges. Firstly, combining metrics with a simple calculation (mean, median or sum) assumes that declines in one metric (e.g. population of a target species) can be balanced by increases in another (e.g. species richness), with no change in the overall value of the combined metric [47]. Such trade-offs mask strong value judgements (for example, increasing species richness could harm a particular species of high conservation importance [48]), and could mean the combined metric is not representative of ecosystem health or function. Other ways of aggregating metrics exist [47], where rules can be defined about which metrics can and cannot trade off, but they are likely too complex to be usable in credit methodologies.

Secondly, normalizing metrics so they can be combined onto the same scale using ‘distance to target’ assumes that there exists an ideal target state and that this value can be known. However, biodiversity is dynamic in time and space [49–51] making it difficult to objectively define such a target state, especially in the context of ongoing climate change [52]. The result is that distance-to-target approaches are highly vulnerable to gaming, as low target values mean that relatively small increases in metrics will register as large gains.

### (c) Stage 3: Detecting

#### (i) Methodology decisions

Once methodologies have defined what credits represent (*Stage 1—Framing*), and the metric by which biodiversity is measured (*Stage 2—Quantifying*), they next must define how outcomes will be detected as the project progresses, and how to attribute any change to the investment. This involves demonstrating two things. Firstly, that biodiversity has not degraded (conservation) or has improved (restoration), and secondly, that this outcomes was caused by the project (i.e. is additional). Note that though nearly all credits are sold per hectare, generally biodiversity is measured across the entire site, so detected gains, as described below, are multiplied by the area of the project site to obtain the number of credits.

Restoration credits need to demonstrate that the site has changed for the better, as a result of the investment. All restoration credits use continuous metrics as opposed to binary conditions, and measure the amount of change relative to a baseline; almost always the metric value in the year the project starts^c,f,k^, and, in subsequent issuance periods, the metric value at the end of the last issuance period (figure 4; decision 3a). In one case, this baseline is adjusted based on estimates of how much biodiversity would have declined in the absence of the project^h^. Then, in each issuance period, the change from baseline is calculated (figure 4; decision 3b), and credits are awarded per unit change in the metric (figure 4; decision 3c). For instance, if the metric rises by 10%, 10 credits are issued per hectare^k^.

Conservation credits detect conservation either by demonstrating that the site has not changed, based on the metric, or that a certain condition has been met (figure 4; decision 3d). For those that demonstrate a lack of change^d,e,f,h,k^, the metric is compared to a baseline—the metric value of the site in the year(s) before the project began (figure 4; decision 3e). Generally, a certain window of deviation from the baseline is allowed^d,f,i,k^, for instance, 10%^f^ (figure 4; decision 3f). If the metric value falls below this window, credits are not issued. If it stays within, either a fixed number of credits is issued^b,c,d^ (in some cases, deducting credits depending on how close the metric is to being outside the window^f^) or the amount is based on estimates of how much the site *could* have been degraded if the project had not intervened^k^ (figure 4; decision 3g). Conservation credits based on a binary condition are issued if the binary condition is met^a,b,c,g,l^, the number of credits issued is sometimes determined based on a numeric measure of the quality of the site^a^ (figure 4; decision 3i).

What would have happened without the investment cannot be directly observed, so it must be estimated. Conservation credit methodologies generally avoid explicitly estimating this counterfactual, but attempt to demonstrate additionality based on either evidence of threat^a,b,g^ (for example, only sites that occur in regions of rapid habitat loss can enrol) or of additional management action^b,f^ (figure 4; decision 3h). All restoration credits in our sample use the baseline to represent what the state of biodiversity would have been without the investment (figure 4; decision 3a)—assuming that in the absence of the project, the site would have remained at the baseline metric value.

#### (ii) Challenges

There are two key challenges at the ‘detecting’ stage. Firstly, detecting true change (or lack thereof) in the system to ensure the credits are not simply commodifying noise, and secondly, being confident that the outcomes of the investment are truly additional.

##### Commodifying noise

In ecology, measurement error is often very large. Though methods to account for imperfect detectability exist (e.g. occupancy modelling and rarefaction curves), these only reduce uncertainty to a certain extent [53,54]. Newer technologies such as acoustic monitoring and camera traps may bring down survey costs, but are also associated with substantial uncertainty [55,56]. Even if it were possible to measure parameters perfectly, most metrics fluctuate through time; for example, trends in butterfly populations can only be detected from long time series because of large interannual variation [57]. As a result, credit methodologies based on changes over short to medium time scales run the risk of rewarding, or penalizing, project operators based on measurement error or random fluctuations—in essence, commodifying noise. At the time of writing, only a few methodologies included stipulations to attempt to account for this^c,d,f,k^ (see decision 3j; electronic supplementary material, table S4).

##### Additionality

Just because a biodiversity change is observed, it does not mean that it was caused by the investment. For this, an estimate of the counterfactual is needed [58] (i.e. how much nature would have been lost, or regeneration occurred, without the investment). Not all sites supposedly threatened with habitat loss really are [32], and many degraded sites are undergoing natural regeneration following land abandonment [59]. Biodiversity credits, like many conservation incentive schemes, are vulnerable to adverse selection—where the landowners most likely to enrol are those with the lowest opportunity costs for carrying out conservation or restoration [60,61]. This incentivizes projects where the conservation or restoration is not additional [61–63].

Biodiversity credits face even greater challenges in demonstrating additionality than the voluntary carbon market due to the lack of data. In the carbon market, the high spatial and temporal resolution of forest change data means that sophisticated methods can be applied to estimate the counterfactual [32,63,64]. However, data on other biodiversity outcomes are not available from remote sensing, and would otherwise be prohibitively costly to collect. For this reason, few credit methodologies use control sites, and those that do use them take static one-time measurements to represent what the project site could be, rather than using a dynamic counterfactual measured through time alongside the project site [65].

One school of thought argues that even if it is difficult or impossible to demonstrate that biodiversity outcomes are additional, the very fact that additional finances have been brought to the project indicates a positive outcome (financial additionality). However additional investment, in and of itself, does not ensure positive biodiversity outcomes, especially as in the past biodiversity markets have induced ‘cost-shifting’—where states use the revenues generated by biodiversity markets to justify reducing their own public biodiversity spending [65].

### (d) Stage 4: Adjusting

#### (i) Methodology decisions

After the number of credits generated has been calculated (*Stage 3—Detecting*), some methodologies adjust the number of credits issued to account for leakage, other uncertainties or permanence.

Leakage is where an investment results in threats to biodiversity being displaced, rather than reduced [66]. To account for it, some methodologies subtract a proportion of credits from the total generated^c,f,g,h,k^ (figure 5; decision 4a). Most only consider leakage at a very local scale, e.g. into areas that are within a project’s control but not being used for biodiversity credits^c,h,k^. None account for leakage over larger distances. Some methodologies also choose to deduct a ‘buffer’, a set percentage (most often 20%^d,f,h,k^), to account for uncertainties^a,c,d,f,h,k^ (figure 5; decision 4b).

Most have stipulations about the minimum length projects must exist for, in order to address the issue of permanence (figure 5; decision 4c), even if credits can be issued at multiple time points within that period. Typically, this is at least 20 years^b,f,g,h,i,k,l^.

#### (ii) Challenges

##### Leakage

Leakage can be direct (sometimes termed primary or local leakage) or market (also known as secondary or indirect leakage) [66]. Direct leakage refers to the direct displacement of threats; for example, a farmer restores one field to generate biodiversity credits but production moves to an undeveloped area elsewhere. Market leakage is mediated through supply chains; for example, if production of a crop reduces because farmers in one place decide to use the land for biodiversity credits, this decrease in supply may cause prices to rise, which incentivizes others elsewhere to develop land to grow the crop.

Though some methodologies include clauses to account for direct leakage, most are vague about how exactly it should be calculated. Previous studies of conservation projects that have tested for the presence of direct leakage (e.g. [67]) have only been able to do so because of the availability of remotely sensed data on forest change. Assessing direct leakage using the detailed biodiversity metrics proposed by credit methodologies will be extremely challenging as it would require biodiversity monitoring across the area producing credits and any land into which leakage may occur.

Even more difficult to assess is market leakage because of teleconnections—distant, often global-scale—links between drivers of demand and local land use [68]. Some economists argue that conservation projects that alter production by using land for biodiversity rather than crops, timber, etc., will always result in leakage because demand must be met from elsewhere [66]. This suggests that the only way to ensure no leakage is to couple conservation/restoration efforts with schemes that either reduce demand for the commodity or promote sustainable intensification so that demand can be met with less land [66,69]. Approaches do exist to estimate the extent of market leakage [68,69], but these are likely to be unfeasible at the project scale.

##### Permanence

It has long been recognized that carbon sequestered through forest conservation or restoration projects does not represent the permanent removal of carbon from the atmosphere, as those forests may be cleared in future [70]. Wherever biodiversity credits are used to offset losses then the question of permanence is just as important; without long-term management, many conservation or restoration gains could be lost. Though many operators specify minimum project lengths, the efficacy of the projects issuing credits remains untested in any nature market because it requires observation over longer time scales. Doubts exist about the robustness of current governance mechanisms for ensuring long-term nature protection [71], not least given the current worsening of major drivers of biodiversity loss such as climate change.

##### Buffer

Setting aside a proportion of credits to act as a buffer against unexpected events is one way to address potential impermanence, as well as market leakage, a lack of additionality and other uncertainties, and has been used extensively in carbon markets. The related concept of offset multipliers has served the same purpose in biodiversity offsetting schemes [72]. However, both have been criticized for setting these at levels that severely underestimate potential uncertainties [31,73]. This would suggest a need instead to explicitly recognize and manage uncertainties [74] as the buffering approach is unlikely to be financially viable if it is appropriately precautionary.

## 3. Discussion

### (a) Confronting ‘deep uncertainty’ in a unit of nature

Georgina Mace made it clear over a decade ago that a phenomenon as complex as biodiversity can never be reduced to a single measure and will always require different metrics for different purposes [8]. The search for ‘one true metric’ is inherently futile. The ‘basket of metrics’ approach adopted by many methodologies does not change this fact, as it involves subjective decisions regarding which metrics are included in the basket and how they are normalized, weighted and aggregated.

Reducing uncertainty in the measurement of biodiversity will be important to ensuring that credits do not reward or penalize projects based on measurement error (‘commodifying noise’). Recent advances in monitoring technology could go some way towards reducing some measurements [75,76], although, at least in the near future, the cost of rolling out such technologies across many project sites is likely to be prohibitive and would reduce the funding available for conservation or restoration activities. Recognizing this, a few credit operators, not included in our full review, do instead reward management action, rather than investing in the monitoring of outcomes^m,n,o,p^ [77].

Better measurement will still not address important issues in operationalizing biodiversity credits, which include the long-recognized difficulties of demonstrating additionality, dealing with leakage and the permanence of any gains. These have been extensively discussed in relation to other schemes providing conditional funding based on conservation outcomes such as payments for ecosystem services (conditional payments incentivizing land managers to provide ecosystem services [78]) and REDD+ (then envisaged as a global-scale framework of government-to-government investment in forest conservation with social and biodiversity co-benefits [79]). Yet despite decades of work, there remains ‘deep uncertainty’ (uncertainty which cannot be known or quantified) associated with such schemes and how they demonstrate truly additional, permanent gains [80].

The uncertainty associated with biodiversity credits will inevitably be even deeper because of the metric difficulties discussed in this article. This is not to mention the potential for perverse actors to intentionally game the system, for example, by selecting metrics likely to show positive change but that poorly represent ecosystem health or local values, or by selecting low values for the ‘target ecosystem’ that metrics are normalized against, to inflate estimated gains. Gaming has been a problem in carbon markets [81], and in previous cases of biodiversity offsetting [82].

All of the above means that knowing whether the investments which biodiversity credits claim to bring to biodiversity conservation are actually having the desired effect will be supremely challenging.

### (b) Risks of biodiversity trading

Proponents of financial additionality argue that all investment in biodiversity is welcome, regardless of uncertainty or whether outcomes are demonstrably additional. In some cases this could seem sensible: a long-established conservation project, seeking additional revenue from conservation credits, could struggle to demonstrate ‘avoided-loss’ if years of efforts have already reduced imminent threats in the area (limiting investment to the exact projects most well positioned to upscale conservation work). However, any situation where there is a gap between claimed and measurable benefits carries risks. First and foremost, if underperforming credits are used by businesses to offset harms, there will be a net loss of biodiversity. Underperforming credits could also crowd out other sources of funding because of the perception that biodiversity loss is being sufficiently addressed, or undermine the long-term credibility and stability of funding flows if it becomes apparent that improvements are not real [19,60].

The commodification of public goods can also risk exacerbating inequality [83]. The development of credits in places where Indigenous peoples and local communities lack secure land rights and are marginalized from decision-making can result in further marginalization and economic displacement [84]. Safeguards developed to address such issues in biodiversity offsets and REDD+ schemes have had mixed success [85,86]. Initiatives have been set up to improve the representation of Indigenous peoples and local communities in the development of biodiversity credits [87,88], but substantial attention and resources will be needed to ensure that biodiversity credits enhance rather than undermine human rights and equity.

Finally, some state that biodiversity is so incompatible with commodification that to compromise by allowing it to be traded is inherently harmful and will result in conservation goals increasingly being compromised by businesses in pursuit of profit [89,90]. Under this reasoning, market-based instruments deplete the political capital required for alternative approaches to funding conservation such as cutting subsidies, increasing public investment, mandating industry contributions to global funds for biodiversity and tightening regulations that address the underlying drivers of biodiversity loss [91].

### (c) Ways forward for biodiversity credits

Given these risks, and the deep uncertainty inherent in creating a tradeable unit of nature, what is the way forward? The key question may not be ‘how do we better define a unit of nature?’, but rather ‘(how) can biodiversity credit markets be designed so that the benefits outweigh the harms?’ This shifts the focus out of the realm of ecology and into the realm of political science, governance and economics.

Using biodiversity credits to quantify contributions toward nature recovery, rather than to directly offset specific negative impacts, is a key way to reduce some of the risks we highlight. This is referred to in the forest carbon world as a ‘contribution’ model. Instead of buyers of forest carbon credits claiming that the credits can offset emissions to achieve ‘net zero’, they instead make a ‘contribution’ to global climate mitigation through investments in forests [92]. While this may seem like a small change in terminology, it represents an important difference. If carbon credits cannot be subtracted from a company’s emissions to produce a single net number, they cannot be used as a license to continue emitting. This also lessens the incentive for buyers to focus on quantity rather than quality in purchased credits [17,93]. Some biodiversity credit operators are already promoting this approach.^e^

However, it is important that the contribution approach does not entrench the idea that an organization just needs to make some level of investment in biodiversity conservation in order to claim a ‘nature positive’ contribution [94]. Genuine application of the mitigation hierarchy is required, emphasizing first and foremost avoiding negative biodiversity impacts [19].

With these caveats, and as long as biodiversity credit investment does not replace public policy effort, and as long as Indigenous peoples and local communities hold the decision-making power regarding whether and how projects go ahead [95], a contributions-based biodiversity credits market could be positive for nature. However, these are very strong suppositions that have not always held in the past: there is ample evidence of the mitigation hierarchy being ignored and harms to Indigenous peoples and local communities from previous schemes [65,96–98].

Regulation could address some of these risks, but effective enforcement of regulation on the voluntary biodiversity credit market would require very substantial industry-generated will, as well as transparency and civil society scrutiny. Even in national-level schemes, which require biodiversity to be measured in a standardized way to meet statutory requirements, such levels of regulatory capacity are rare [26–28].

## 4. Conclusion

Developing standardized approaches to measure units of biodiversity is a monumental challenge. Any attempt to abstract nature needs to be transparent about the ways in which units can, and cannot, represent biodiversity and its values. We hope that our analysis of the stages that biodiversity credit methodologies must go through to produce a tradeable unit has highlighted key challenges and the very substantial uncertainties. Based on this analysis, we believe that a positive impact from credits is only possible in a ‘contribution’ model [92], where credits are used to make a measurable contribution to biodiversity, rather than being used to offset negative impacts in support of ‘net’ claims.

Progress towards humanity’s urgent mission to halt and reverse the loss of biodiversity depends upon the cumulative actions of all sectors of society. There may be a role for markets in innovating, and reaching places, actors and funding that would otherwise be unreachable. However, markets can only ever be one part of the solution for delivering effective and equitable conservation, and there will remain a large and important role for direct investment in nature by the public and private sectors [99] as well as for regulation to reduce impacts on nature. This will be especially vital to ensuring the conservation and recovery of the aspects of nature, and its importance for people, that cannot be reduced to a unit.

## Data Availability

Supplementary material is available online [100].

## References

[1] Mace GM, Lande R. 1991 Assessing extinction threats: toward a reevaluation of IUCN threatened species categories. Conserv. Biol. **5**, 148–157. (10.1111/j.1523-1739.1991.tb00119.x)

[2] Mace GM, Collar NJ, Gaston KJ, Hilton-Taylor C, Akçakaya HR, Leader-Williams N, Milner-Gulland EJ, Stuart SN. 2008 Quantification of extinction risk: IUCN’s system for classifying threatened species. Conserv. Biol. **22**, 1424–1442. (10.1111/j.1523-1739.2008.01044.x) 18847444

[3] Hockings M *et al*. 2019 The IUCN Green List of Protected and Conserved Areas: setting the standard for effective conservation. PARKS **25**, 57–66. (10.2305/IUCN.CH.2019.PARKS-25-2MH.en)

[4] Grace MK *et al*. 2021 Testing a global standard for quantifying species recovery and assessing conservation impact. Conserv. Biol. **35**, 1833–1849. (10.1111/cobi.13756) 34289517

[5] Watson R, Baste I, Larigauderie A *et al*. 2019 Summary for policymakers of the global assessment report on biodiversity and ecosystem services of the intergovernmental science-policy platform on biodiversity and ecosystem services, pp. 22–47. Bonn, Germany: IPBES Secretariat.

[6] Watson RT. 2012 The science-policy interface: the role of scientific assessments: UK national ecosystem assessment. Proc. Math. Phys. Eng. Sci. **468**, 3265–3281. (10.1098/rspa.2012.0163) 23197933 PMC3509955

[7] Bateman IJ *et al*. 2013 Bringing ecosystem services into economic decision-making: land use in the United Kingdom. Science **341**, 45–50. (10.1126/science.1234379) 23828934

[8] Mace GM, Norris K, Fitter AH. 2012 Biodiversity and ecosystem services: a multilayered relationship. Trends Ecol. Evol. **27**, 19–26. (10.1016/j.tree.2011.08.006) 21943703

[9] Baillie JEM *et al*. 2008 Toward monitoring global biodiversity. Conserv. Lett. **1**, 18–26. (10.1111/j.1755-263X.2008.00009.x)

[10] Mace GM, Barrett M, Burgess ND, Cornell SE, Freeman R, Grooten M, Purvis A. 2018 Aiming higher to bend the curve of biodiversity loss. Nat. Sustain. **1**, 448–451. (10.1038/s41893-018-0130-0)

[11] Leclère D *et al*. 2020 Bending the curve of terrestrial biodiversity needs an integrated strategy. Nature **585**, 551–556. (10.1038/s41586-020-2705-y) 32908312

[12] Obura DO *et al*. 2023 Achieving a nature- and people-positive future. One Earth **6**, 105–117. (10.1016/j.oneear.2022.11.013)

[13] Burgess ND *et al*. 2024 Global metrics for terrestrial biodiversity. Annu. Rev. Environ. Resour. **49**, 673–709. (10.1146/annurev-environ-121522-045106)

[14] Christie M, Hanley N, Warren J, Murphy K, Wright R, Hyde T. 2006 Valuing the diversity of biodiversity. Ecol. Econ. **58**, 304–317. (10.1016/j.ecolecon.2005.07.034)

[15] Purvis A, Hector A. 2000 Getting the measure of biodiversity. Nature **405**, 212–219. (10.1038/35012221) 10821281

[16] Biodiversity Credit Alliance. 2024 Definition of a biodiversity credit. See https://www.biodiversitycreditalliance.org/wp-content/uploads/2024/05/Definition-of-a-Biodiversity-Credit-Rev-220524.pdf.

[17] Taskforce on Nature Markets. 2023 Biodiversity credit markets. https://www.naturefinance.net/wp-content/uploads/2023/04/BiodiversityCreditMarkets.pdf

[18] Salzman J, Ruhl JB. 2000 Currencies and the commodification of environmental law. Stanford Law Rev. **53**, 607. (10.2307/1229470)

[19] Maron M *et al*. 2024 'Nature positive' must incorporate, not undermine, the mitigation hierarchy. Nat. Ecol. Evol. **8**, 14–17. (10.1038/s41559-023-02199-2) 37735564

[20] Bull JW, Strange N. 2018 The global extent of biodiversity offset implementation under no net loss policies. Nat. Sustain. **1**, 790–798. (10.1038/s41893-018-0176-z)

[21] Koh NS, Hahn T, Boonstra WJ. 2019 How much of a market is involved in a biodiversity offset? A typology of biodiversity offset policies. J. Environ. Manage. **232**, 679–691. (10.1016/j.jenvman.2018.11.080) 30522073

[22] United Nations Environment Programme. State of finance for nature 2023: the big nature turnaround—repurposing $7 trillion to combat nature loss. Nairobi: United Nations Environment Programme. (10.59117/20.500.11822/44278)

[23] Damiens FLP, Porter L, Gordon A. 2021 The politics of biodiversity offsetting across time and institutional scales. Nat. Sustain. **4**, 170–179. (10.1038/s41893-020-00636-9)

[24] Needham K, de Vries FP, Armsworth PR, Hanley N. 2019 Designing markets for biodiversity offsets: lessons from tradable pollution permits. J. Appl. Ecol. **56**, 1429–1435. (10.1111/1365-2664.13372)

[25] International Advisory Panel on Biodiversity Credits. 2024 IAPB consultation on archetypes: executive summary. https://www.iapbiocredits.org/resources.

[26] Theis S, Ruppert JLW, Roberts KN, Minns CK, Koops M, Poesch MS. 2020 Compliance with and ecosystem function of biodiversity offsets in North American and European freshwaters. Conserv. Biol. **34**, 41–53. (10.1111/cobi.13343) 31058355

[27] Evans MC. 2023 Backloading to extinction: coping with values conflict in the administration of Australia’s federal biodiversity offset policy. Aust. J. Public Adm. **82**, 228–247. (10.1111/1467-8500.12581)

[28] Rampling EE, Zu Ermgassen SOSE, Hawkins I, Bull JW. 2024 Achieving biodiversity net gain by addressing governance gaps underpinning ecological compensation policies. Conserv. Biol. **38**, e14198. (10.1111/cobi.14198) 37811729

[29] zu Ermgassen S, Baker J, Griffiths RA, Strange N, Struebig MJ, Bull JW. 2019 The ecological outcomes of biodiversity offsets under 'no net loss' policies: a global review. Conserv. Lett. **12**, e12664. (10.1111/conl.12664)

[30] Josefsson J, Widenfalk LA, Blicharska M, Hedblom M, Pärt T, Ranius T, Öckinger E. 2021 Compensating for lost nature values through biodiversity offsetting – where is the evidence? Biol. Conserv. **257**, 109117. (10.1016/j.biocon.2021.109117)

[31] Badgley G, Freeman J, Hamman JJ, Haya B, Trugman AT, Anderegg WRL, Cullenward D. 2022 Systematic over-crediting in California’s forest carbon offsets program. Glob. Chang. Biol. **28**, 1433–1445. (10.1111/gcb.15943) 34668621 PMC9299598

[32] West TAP, Wunder S, Sills EO, Börner J, Rifai SW, Neidermeier AN, Frey GP, Kontoleon A. 2023 Action needed to make carbon offsets from forest conservation work for climate change mitigation. Science **381**, 873–877. (10.1126/science.ade3535) 37616370

[33] Procton A. 2024 State of the voluntary carbon market 2024. on the path to maturity. Washington, DC: Forest Trends’ Ecosystem Marketplace.

[34] Wunder S *et al*. 2024 Biodiversity credits: learning lessons from other approaches to incentivize conservation. OSF Preprints. (10.31219/osf.io/qgwfc)

[35] James SP. 2022 How nature matters: culture, identity and environmental value. Oxford, UK: Oxford University Press.

[36] Walker S, Brower AL, Stephens RTT, Lee WG. 2009 Why bartering biodiversity fails. Conserv. Lett. **2**, 149–157. (10.1111/j.1755-263X.2009.00061.x)

[37] Gao S, Bull JW, Baker J, zu Ermgassen SOSE, Milner‐Gulland EJ. 2023 Analyzing the outcomes of China’s ecological compensation scheme for development‐related biodiversity loss. Conservat. Sci. and Prac. **5**, e13010. (10.1111/csp2.13010)

[38] zu Ermgassen SOSE, Marsh S, Ryland K, Church E, Marsh R, Bull JW. 2021 Exploring the ecological outcomes of mandatory biodiversity net gain using evidence from early‐adopter jurisdictions in England. Conserv. Lett. **14**, e12820. (10.1111/conl.12820)

[39] Burgass MJ, Halpern BS, Nicholson E, Milner-Gulland EJ. 2017 Navigating uncertainty in environmental composite indicators. Ecol. Indic. **75**, 268–278. (10.1016/j.ecolind.2016.12.034)

[40] Nardo M, Saisana M, Saltelli A, Tarantola S, Hoffman A, Giovannini E. 2005 Handbook on constructing composite indicators: methodology and user guide. Organisation for Economic Co-operation and Development. (10.1787/533411815016)

[41] Buckland ST, Magurran AE, Green RE, Fewster RM. 2005 Monitoring change in biodiversity through composite indices. Philos. Trans. R. Soc. Lond. B Biol. Sci. **360**, 243–254. (10.1098/rstb.2004.1589) 15814343 PMC1569463

[42] Cuff JP, Deivarajan Suresh M, Dopson MEG, Hawthorne BSJ, Howells T, Kitson JJN, Miller KA, Xin T, Evans DM. 2023 Chapter one - a roadmap for biomonitoring in the 21st century: merging methods into metrics via ecological networks. Adv. Ecol. Res. (eds DA Bohan, AJ Dumbrell), **68**, 1–34. (10.1016/bs.aecr.2023.09.002)

[43] Luque-Lora R. 2023 The trouble with relational values. Environ. Values **32**, 411–431. (10.3197/096327122X16611552268681)

[44] Biodiversity Credit Alliance. 2023 Demand-side sources and motivation for biodiversity credits. Biodiversity Credit Alliance.

[45] Deere NJ, Ramadiyanta E, Sibarani MC, Hadi AN, Andayani N, Ginting Y, Bull JW, Struebig MJ. 2024 Selecting umbrella species as mammal biodiversity indicators in tropical forest. Biol. Conserv. **292**, 110511. (10.1016/j.biocon.2024.110511)

[46] Deere NJ *et al*. 2020 Implications of zero‐deforestation commitments: forest quality and hunting pressure limit mammal persistence in fragmented tropical landscapes. Conserv. Lett. **13**, e12701. (10.1111/conl.12701)

[47] Greco S, Ishizaka A, Tasiou M, Torrisi G. 2019 On the methodological framework of composite indices: a review of the issues of weighting, aggregation, and robustness. Soc. Indic. Res. **141**, 61–94. (10.1007/s11205-017-1832-9)

[48] O’Neill J. 2017 Pluralism and incommensurability. In Routledge handbook of ecological economics (ed. CL Spash), pp. 227–236. Abingdon, UK: Routledge. (10.4324/9781315679747-28)

[49] Trisos CH, Merow C, Pigot AL. 2020 The projected timing of abrupt ecological disruption from climate change. Nature **580**, 496–501. (10.1038/s41586-020-2189-9) 32322063

[50] Jones C, Lowe J, Liddicoat S, Betts R. 2009 Committed terrestrial ecosystem changes due to climate change. Nat. Geosci. **2**, 484–487. (10.1038/ngeo555)

[51] Dornelas M, Gotelli NJ, Shimadzu H, Moyes F, Magurran AE, McGill BJ. 2019 A balance of winners and losers in the anthropocene. Ecol. Lett. **22**, 847–854. (10.1111/ele.13242) 30874368

[52] Westwood A, Reuchlin-Hugenholtz E, Keith DM. 2014 Re-defining recovery: a generalized framework for assessing species recovery. Biol. Conserv. **172**, 155–162. (10.1016/j.biocon.2014.02.031)

[53] Gwinn DC, Allen MS, Bonvechio KI, V. Hoyer M, Beesley LS. 2016 Evaluating estimators of species richness: the importance of considering statistical error rates. Methods Ecol. Evol. **7**, 294–302. (10.1111/2041-210X.12462)

[54] Kowalewski LK, Chizinski CJ, Powell LA, Pope KL, Pegg MA. 2015 Accuracy or precision: implications of sample design and methodology on abundance estimation. Ecol. Modell. **316**, 185–190. (10.1016/j.ecolmodel.2015.08.016)

[55] Wood CM, Kahl S, Chaon P, Peery MZ, Klinck H. 2021 Survey coverage, recording duration and community composition affect observed species richness in passive acoustic surveys. Methods Ecol. Evol. **12**, 885–896. (10.1111/2041-210X.13571)

[56] Gilbert NA, Clare JDJ, Stenglein JL, Zuckerberg B. 2021 Abundance estimation of unmarked animals based on camera-trap data. Conserv. Biol. **35**, 88–100. (10.1111/cobi.13517) 32297655

[57] Thomas JA. 2005 Monitoring change in the abundance and distribution of insects using butterflies and other indicator groups. Phil. Trans. R. Soc. B **360**, 339–357. (10.1098/rstb.2004.1585) 15814349 PMC1569450

[58] Ferraro PJ. 2009 Counterfactual thinking and impact evaluation in environmental policy. New Drctns. Evaluation **2009**, 75–84. (10.1002/ev.297)

[59] Meli P, Holl KD, Rey Benayas JM, Jones HP, Jones PC, Montoya D, Moreno Mateos D. 2017 A global review of past land use, climate, and active vs. passive restoration effects on forest recovery. PLoS One **12**, e0171368. (10.1371/journal.pone.0171368) 28158256 PMC5291368

[60] Swinfield T, Shrikanth S, Bull JW, Madhavapeddy A, zu Ermgassen SOSE. 2024 Nature-based credit markets at a crossroads. Nat. Sustain. **7**, 1217–1220. (10.1038/s41893-024-01403-w)

[61] Jack BK, Jayachandran S. 2019 Self-selection into payments for ecosystem services programs. Proc. Natl Acad. Sci. USA **116**, 5326–5333. (10.1073/pnas.1802868115) 30072433 PMC6431215

[62] Zu Ermgassen SOSE *et al*. 2023 Evaluating the impact of biodiversity offsetting on native vegetation. Glob. Chang. Biol. **29**, 4397–4411. (10.1111/gcb.16801) 37300408 PMC10946555

[63] Macintosh A *et al*. 2024 Australian human-induced native forest regeneration carbon offset projects have limited impact on changes in woody vegetation cover and carbon removals. Commun. Earth Environ. **5**. (10.1038/s43247-024-01313-x)

[64] Guizar-Coutiño A, Jones JPG, Balmford A, Carmenta R, Coomes DA. 2022 A global evaluation of the effectiveness of voluntary redd+ projects at reducing deforestation and degradation in the moist tropics. Conserv. Biol. **36**, e13970. (10.1111/cobi.13970) 35713105 PMC10086997

[65] Narain D, Maron M. 2018 Cost shifting and other perverse incentives in biodiversity offsetting in India. Conserv. Biol. **32**, 782–788. (10.1111/cobi.13100) 29473220

[66] Filewod B, McCarney G. 2023 Avoiding carbon leakage from nature-based offsets by design. One Earth **6**, 790–802. (10.1016/j.oneear.2023.05.024)

[67] Devenish K, Desbureaux S, Willcock S, Jones JPG. 2022 On track to achieve no net loss of forest at Madagascar’s biggest mine. Nat. Sustain. **5**, 498–508. (10.1038/s41893-022-00850-7)

[68] Henders S, Ostwald M. 2014 Accounting methods for international land-related leakage and distant deforestation drivers. Ecol. Econ. **99**, 21–28. (10.1016/j.ecolecon.2014.01.005)

[69] Streck C. 2021 REDD+ and leakage: debunking myths and promoting integrated solutions. Clim. Pol. **21**, 843–852. (10.1080/14693062.2021.1920363)

[70] Marland G, Fruit K, Sedjo R. 2001 Accounting for sequestered carbon: the question of permanence. Environ. Sci. Policy **4**, 259–268. (10.1016/S1462-9011(01)00038-7)

[71] Damiens FLP, Backstrom A, Gordon A. 2021 Governing for 'no net loss' of biodiversity over the long term: challenges and pathways forward. One Earth **4**, 60–74. (10.1016/j.oneear.2020.12.012)

[72] Moilanen A, Van Teeffelen AJA, Ben‐Haim Y, Ferrier S. 2009 How much compensation is enough? A framework for incorporating uncertainty and time discounting when calculating offset ratios for impacted habitat. Restor. Ecol. **17**, 470–478. (10.1111/j.1526-100X.2008.00382.x)

[73] Laitila J, Moilanen A, Pouzols FM. 2014 A method for calculating minimum biodiversity offset multipliers accounting for time discounting, additionality and permanence. Methods Ecol. Evol. **5**, 1247–1254. (10.1111/2041-210X.12287) 25821578 PMC4374704

[74] Milner-Gulland EJ, Shea K. 2017 Embracing uncertainty in applied ecology. J. Appl. Ecol. **54**, 2063–2068. (10.1111/1365-2664.12887) 29225369 PMC5722456

[75] Navarro LM *et al*. 2017 Monitoring biodiversity change through effective global coordination. Curr. Opin. Environ. Sustain. **29**, 158–169. (10.1016/j.cosust.2018.02.005)

[76] Ford HV, Schrodt F, Zieritz A, Exton DA, van der Heijden G, Teague J, Coles T, Field R. 2024 A technological biodiversity monitoring toolkit for biocredits. J. Appl. Ecol. **61**, 2007–2019. (10.1111/1365-2664.14725)

[77] Gibbons JM, Nicholson E, Milner‐Gulland EJ, Jones JPG. 2011 Should payments for biodiversity conservation be based on action or results? J. Appl. Ecol. **48**, 1218–1226. (10.1111/j.1365-2664.2011.02022.x)

[78] Wunder S. 2007 The efficiency of payments for environmental services in tropical conservation. Conserv. Biol. **21**, 48–58. (10.1111/j.1523-1739.2006.00559.x) 17298510

[79] Phelps J, Webb EL, Agrawal A. 2010 Land use: does REDD+ threaten to recentralize forest governance? Science **328**, 312–313. (10.1126/science.1187774) 20395498

[80] Wells G, Pascual U, Stephenson C, Ryan CM. 2023 Confronting deep uncertainty in the forest carbon industry. Science **382**, 41–43. (10.1126/science.adh8117) 37796998

[81] Aguilar-Støen M. 2017 Better safe than sorry? Indigenous peoples, carbon cowboys and the governance of redd in the amazon. Forum Dev. Stud. **44**, 91–108. (10.1080/08039410.2016.1276098)

[82] Maron M, Bull JW, Evans MC, Gordon A. 2015 Locking in loss: baselines of decline in Australian biodiversity offset policies. Biol. Conserv. **192**, 504–512. (10.1016/j.biocon.2015.05.017)

[83] Christophers B. 2023 Our lives in their portfolios: why asset managers own the world. London, UK: Verso.

[84] Young D. 2024 Beyond offsets: people and planet-centred responses to the climate and biodiversity crisis. London, UK: Rainforest Foundation UK.

[85] Bidaud C, Schreckenberg K, Jones JPG. 2018 The local costs of biodiversity offsets: comparing standards, policy and practice. Land use policy **77**, 43–50. (10.1016/j.landusepol.2018.05.003)

[86] Poudyal M, Ramamonjisoa BS, Hockley N, Rakotonarivo OS, Gibbons JM, Mandimbiniaina R, Rasoamanana A, Jones JPG. 2016 Can redd+ social safeguards reach the ‘right’ people? lessons from Madagascar. Glob. Environ. Change. **37**, 31–42. (10.1016/j.gloenvcha.2016.01.004)

[87] Gorring A, Hutchinson J. 2023 Leading for nature. https://pollinationgroup.com/wp-content/uploads/2024/02/Leading-for-Nature-Report.pdf

[88] Biodiversity Credit Alliance. 2023 Communities and nature markets: building just partnerships in biodiversity credits. Biodiversity Credit Alliance.

[89] Spash CL. 2015 Bulldozing biodiversity: the economics of offsets and trading-in nature. Biol. Conserv. **192**, 541–551. (10.1016/j.biocon.2015.07.037)

[90] Ives CD, Bekessy SA. 2015 The ethics of offsetting nature. Front. Ecol. Environ. **13**, 568–573. (10.1890/150021)

[91] Dempsey J, Suarez DC. 2016 Arrested development? The promises and paradoxes of 'selling nature to save it'. Ann. Am. Assoc. Geogr. **106**, 653–671. (10.1080/24694452.2016.1140018)

[92] Blanchard L *et al*. 2024 Funding forests’ climate potential without carbon offsets. One Earth **7**, 1147–1150. (10.1016/j.oneear.2024.06.006)

[93] Trencher G, Nick S, Carlson J, Johnson M. 2024 Demand for low-quality offsets by major companies undermines climate integrity of the voluntary carbon market. Nat. Commun. **15**, 6863. (10.1038/s41467-024-51151-w) 39127784 PMC11316763

[94] Milner-Gulland EJ. 2022 Don’t dilute the term nature positive. Nat. Ecol. Evol. **6**, 1243–1244. (10.1038/s41559-022-01845-5) 35941204

[95] Cariño J, Ferrari MF. 2021 Negotiating the futures of nature and cultures: perspectives from indigenous peoples and local communities about the post-2020 global biodiversity framework. J. Ethnobiol. **41**, 192–208. (10.2993/0278-0771-41.2.192)

[96] Phalan B, Hayes G, Brooks S, Marsh D, Howard P, Costelloe B, Vira B, Kowalska A, Whitaker S. 2018 Avoiding impacts on biodiversity through strengthening the first stage of the mitigation hierarchy. Oryx **52**, 316–324. (10.1017/S0030605316001034)

[97] Clare S, Krogman N, Foote L, Lemphers N. 2011 Where is the avoidance in the implementation of wetland law and policy? Wetl. Ecol. Manag. **19**, 165–182. (10.1007/s11273-011-9209-3)

[98] Dunne D, Quiroz Y. 2023 Mapped: the impacts of carbon-offset projects around the world. See https://interactive.carbonbrief.org/carbon-offsets-2023/mapped.html.

[99] Kedward K, Zu Ermgassen S, Ryan-Collins J, Wunder S. 2023 Heavy reliance on private finance alone will not deliver conservation goals. Nat. Ecol. Evol. **7**, 1339–1342. (10.1038/s41559-023-02098-6) 37308705

[100] Wauchope HS, zu Ermgassen S, Jones JPG, Carter H, Schulte to Bühne H, Milner-Gulland EJ. 2024 Supplementary material from: What is a unit of nature? Measurement challenges in the emerging biodiversity credit market. Figshare. (10.6084/m9.figshare.c.7539182) PMC11631508 39657801

